# Hormonal, functional and genetic biomarkers in controlled ovarian stimulation: tools for matching patients and protocols

**DOI:** 10.1186/1477-7827-10-9

**Published:** 2012-02-06

**Authors:** Carlo Alviggi, Peter Humaidan, Diego Ezcurra

**Affiliations:** 1 Centro di Sterilità ed Infertilità di Coppia, Università degli Studi di Napoli "Federico II", Naples, Italy; 2 The Fertility Clinic, The University Hospital Odense (OUH), Denmark; 3 Fertility and Endocrinology Business Unit, Merck Serono S.A., Geneva, Switzerland

**Keywords:** controlled ovarian stimulation, biomarkers, personalised, genetic screening

## Abstract

Variability in the subfertile patient population excludes the possibility of a single approach to controlled ovarian stimulation (COS) covering all the requirements of a patient. Modern technology has led to the development of new drugs, treatment options and quantitative methods that can identify single patient characteristics. These could potentially be used to match patients with the right treatment options to optimise efficacy, safety and tolerability during COS. Currently, age and follicle-stimulating hormone (FSH) level remain the most commonly used single patient characteristics in clinical practice. These variables only provide a basic prognosis for success and indications for standard COS treatment based on gross patient categorisation. In contrast, the anti-Müllerian hormone level appears to be an accurate predictor of ovarian reserve and response to COS, and could be used successfully to guide COS. The antral follicle count is a functional biomarker that could be useful in determining the dose of FSH necessary during stimulation and the success of treatment. Finally, in the future, genetic screening may allow an individual patient's response to stimulation during COS to be predicted based on genotype. Unfortunately, despite the predictive power of these measures, no single biomarker can stand alone as a guide to determine the best treatment option. In the future, hormonal, functional and genetic biomarkers will be used together to personalise COS.

## Background

Modern medical science has made great advances in the understanding and treatment of subfertility. This review is not an evidence-based meta-analysis, but rather provides our opinion on what we foresee as the future for controlled ovarian stimulation (COS) and how treatment protocols could be optimised to improve the outcome for individual patients. It is worth noting that some of the studies available in the literature and reported here have limited sample sizes and may not be adequately powered to show significance.

Novel technologies have created new ways to evaluate and treat patients and provide them with a prognosis for overcoming their subfertility. Basic and clinical research coupled with advances in technology have led to the development of a wide variety of new treatment options, particularly in the area of COS. Follicle-stimulating hormone (FSH [urinary or recombinant (u-FSH/r-FSH)]) levels can be used alone or associated with luteinising hormone (LH) activity, which in turn can be provided in the form of human menopausal gonadotrophin (hMG), recombinant human LH (r-hLH) or human chorionic gonadotrophin (hCG [urinary or recombinant (u-hCG/r-hCG)]). In addition, the optional use of steroid hormones such as progesterone, oestrogen, testosterone or dehydroepiandrosterone (DHEA), and even the possibility of growth hormone treatment, could be considered following further research. Taken together, all these options tend to increase the complexity of treatment decisions. The discussion around gonadotrophin-releasing hormone (GnRH) agonist versus antagonist protocols during COS is another example highlighting the expanding number of treatment options and the urgent need to develop a rationale for making the best choice of treatment for each single patient.

Currently, demographic and anthropomorphic data are the primary sources of information used to provide a prognosis and guide treatment. Along with basic medical and nutritional information, functional, hormonal and genetic biomarkers are tools that could provide more accurate diagnostic and prognostic information. Nonetheless, there is currently no consensus in the literature around the world as to how these newly available tools should be applied for a personalised treatment plan in order to improve the outcome of COS. Presently, general patient characteristics determine treatment, disregarding unique characteristics that might influence the needs of the individual patient, and thus, the chance of success. Empirical decision-making based on broad generalisations translates to similar patients receiving similar treatments. Thus, conventional factors like age and previous cycle data are used to make vital treatment decisions. Quantitative biomarkers are utilised mainly to predict prognosis and to inform patient counselling, but the strong potential of these indicators as tools to personalise COS is often underestimated [1].

The multidimensional variability of the subfertile patient population renders a single treatment approach unsuitable to dealing with a wide range of patient characteristics. This diversity in the fertility patient population, together with the ever-expanding number of treatment options (Table 1), creates both the need and the opportunity for personalised treatment, including an individualised approach to COS. The goal of 'personalised COS' can be achieved by taking advantage of the information provided by advancing science and new technologies. Using hormonal, functional and genetic biomarkers to develop a standard method of customising COS will make it possible to consistently match the correct treatment to the right patient. The improvements in treatment decision-making will lead to less burdensome protocols, less risk of adverse events, better outcomes and cost efficiency.

**Table 1:** Variables in the choice of COS protocol

Factors determining individual patients' response to ovarian stimulation	Treatments used in ovarian stimulation protocols	Protocol variations
Demographics and anthropometrics (e.g., age, BMI and race)	Gonadotrophins: r-FSH/LH/hCG u-FSH/LH/hCG	GnRH agonist protocol (long, short and micro flare)
Genetic profile	GnRH analogues: Agonists Antagonists	GnRH antagonist protocol (standard, mild and modified natural)
Health status	Steroid hormones: Progesterone Oestradiol Testosterone DHEA	Agonist-antagonist protocol
Cause of subfertility	Other treatments: Aromatase inhibitors Growth hormone Clomiphene citrate	Natural cycles
Duration of subfertility		
Nutrition		

BMI = body mass index; COS = controlled ovarian stimulation; DHEA = dehydroepiandrosterone; GnRH = gonadotrophin releasing hormone; hCG = human chronic gonadotrophin; LH = luteinising hormone; r-FSH = recombinant follicle-stimulating hormone; u-FSH = urinary follicle-stimulating hormone.

## How can we predict ovarian response and personalise treatment?

### Age versus biomarkers

Age is the most commonly used predictor of fertility; increasing age inevitably results in substantial germ cell loss by the age of 40 [2]. Statistics for pregnancy, live birth and singleton live birth rate also decline rapidly from the mid-thirties on [3]. However, even though age is clearly linked to declining fertility, it does not affect all women equally. On an individual basis, the chronological age of an individual may not be as valuable a predictor of fertility as their 'biological age', which is defined by the hormonal and functional profiles. Thus, biomarkers quantifying hormonal and functional profiles are more useful as prognostic factors than age alone when predicting response and choosing treatment strategy. Finally, genetic screening can provide specific information about a woman's reproductive system that would not be accurately predicted by age, hormonal or functional biomarkers. By considering hormonal (FSH and anti-Müllerian hormone [AMH]), functional (antral follicle count [AFC]), and genetic biomarkers in combination, a complete picture of an individual patient's overall reproductive status can be formulated to provide a basis of designing an optimal treatment plan.

### Hormonal biomarkers: AMH

Inhibin-B, FSH and AMH are hormonal markers that can be used to predict ovarian response to COS and success with assisted reproductive technology (ART). Although FSH levels are more commonly used to evaluate a woman's probable response to COS, AMH (as a paracrine product of immature follicles) is a more direct measure of ovarian status compared with other endocrine reproductive hormones. AMH is primarily produced by the pre-antral and small antral follicles, and correlates with the number of primordial follicles at the gonadotrophin-independent stage of follicular development [4,5].

A small study showing the potential use of AMH measurement in a routine in-vitro fertilisation (IVF) programme prospectively enrolled 316 patients and considered 132 oocyte retrievals in women undergoing a GnRH agonist long protocol. When the calculated optimal AMH cut-off of ≤1.26 ng/ml was used to predict responses to COS, it was found to have a 97% sensitivity for predicting poor responses (< 4 oocytes retrieved) and a 98% accuracy in predicting a normal COS response. These findings indicate that circulating AMH levels may be a good indicator of ovarian reserve, and are highly correlated with ovarian response to COS [6].

In the first study designed to evaluate the use of a hormonal biomarker to determine treatment strategy, Nelson and colleagues compared the safety, tolerability and success rates of different AMH-based COS strategies in two IVF centres. In this prospective cohort study, 538 women were stratified according to their AMH levels prior to COS (low: 1- < 5 pmol/l, medium: 5-15 pmol/l and high: > 15 pmol/l). In both centres, doses were similarly determined by each patient's AMH level; high, medium or low doses of FSH were given to women with low, medium and high AMH levels, respectively. However, in one centre, the choice between the GnRH agonist long protocol versus a milder GnRH antagonist protocol was also determined based on AMH levels. Specifically, most patients at the first centre (regardless of AMH level) were treated with a GnRH agonist long protocol, while in the second centre, those with high or low AMH levels were treated with a GnRH antagonist protocol.

**Figure 1:**
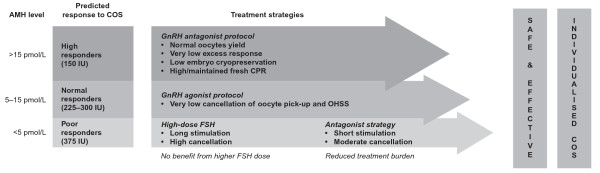
**Treatment strategies and predicted response to COS based on patient circulating AMH levels**. Treatment strategies (right column) and predicted response to COS (middle column) based on patient circulating AMH levels (left column). AMH = anti-Müllerian hormone; COS = controlled ovarian stimulation; GnRH = gonadotrophin releasing hormone; CPR = clinical pregnancy rate; OHSS = ovarian hyperstimulation syndrome; FSH = follicle-stimulating hormone.

In both centres, low AMH levels were associated with a reduced clinical pregnancy rate (CPR), but the cohort of low AMH patients receiving the antagonist protocol had a median duration of treatment of 10 days with a cancellation rate of 5% compared with a duration of 14 days and cancellation rate of 19% for the group treated with GnRH agonist long protocol. As expected, women from both centres with normal AMH levels had similar good outcomes with no poor response and no incidence of excessive stimulation. However, the group of patients with high AMH levels, similar to the group with low AMH levels, had better outcomes using the GnRH antagonist compared with the agonist protocol. No patients with high AMH who underwent the GnRH antagonist protocol were hospitalised due to ovarian hyperstimulation syndrome (OHSS) compared with 13.9% in the high AMH level group who received the GnRH agonist long protocol. In addition, use of the antagonist protocol did not result in any cases of 'total freeze' to avoid OHSS. Furthermore, the improvements in COS safety and tolerability observed with the GnRH antagonist protocol were accompanied by higher CPRs in both the low AMH (11.1% vs 18.7%) and high AMH (40.1% vs 63.6%) groups [7]. The data from this study should be confirmed in large, randomised, controlled trials, but suggest that AMH may be used as a precise and reliable predictive tool to determine treatment strategies that can improve safety, tolerability and pregnancy outcomes (Figure 1).

Currently, a limiting factor in the use of AMH as a biomarker is the variability seen between commercial kits and variation in results reported from different laboratories [8]. This variability may be one of the reasons for the lack of consensus on the cut off values of AMH when deciding whether or not to proceed with ART.

### Functional biomarkers: AFC

The number of antral follicles detected by ovarian ultrasound has gained acceptance as an indicator of ovarian reserve, a COS response prognostic tool and predictor of IVF success [9,10]. In a study including 149 treatment cycles, Chang and colleagues found that AFC declined with age and was negatively correlated to day 3 FSH and cancellation rate. In the same study, when patients were grouped by AFC (AFC < 4, 4-10 or > 10), the mean number of retrieved oocytes was 2.0, 6.3 and 14.0, and ongoing pregnancy rates were 0%, 13.2% and 26.3%, respectively [11].

Another study investigated 113 women undergoing their first ART cycle. In this group, AFC was found to be significantly associated with AMH levels and the number of retrieved oocytes, with the number of follicles between 5 and 6 mm having the highest correlation to both endpoints. AFC was also found to be predictive of poor responses to COS, with the number of follicles sized 2-4 mm, 2-5 mm, 2-8 mm and 2-10 mm being approximately equal in predicting response [12].

AFC is a relatively easy-to-perform technique that is widely accessible due to the availability of ultrasound equipment. Despite its general acceptance as a predictor of ovarian response, the routine use of AFC has been hampered by the lack of a standard methodology that would permit valid comparisons of data from different centres, and thus facilitate the consistent assessment of AFC. In order to address this issue, Broekmans and colleagues suggested recommendations for the standardisation of AFC assessment in routine clinical practice. Basic clinical and technical requirements for AFC evaluation were defined and a systematic method of measuring and counting antral follicles in clinical practice was proposed (Table 2) [13]. Hopefully, as these guidelines are adopted around the world, the accumulation of standardised, universally relevant AFC data will make this quantitative, functional biomarker an even more useful prognostic tool.

**Table 2:** The basic clinical and technical requirements for assessment of the AFC in clinical practice (reproduced with permission from Broekmans et al.

Considerations for the assessment of the AFC in clinical practice	
*Clinical considerations*	*Technical considerations*
Select patients with regular menstrual cycles with no co-existing pathological condition that could technically affect the counting of follicles, such as ovarian endometriosis or previous ovarian surgery	A limited number of personnel, appropriately trained in transvaginal sonography should perform AFCs in each unit Real-time, two-dimensional imaging is adequate
Count follicles between days 2 and 4 of a spontaneous menstrual or oral contraceptive cycle to avoid the effect of intra-cycle variation Include all antral follicles of 2-10 mm in diameter	Use a transvaginal transducer Use a probe with a minimum frequency of 7 MHz, which is maintained in an adequate condition and able to resolve a structure of 2 mm in diameter
	Use a systematic process for counting antral follicles:
	1. Identify the ovary 2. Explore the dimensions in two planes (perform a scout sweep)Decide on the direction of the sweep to measure and count follicles 3. Measure the largest follicle in two dimensions
	A. If the largest follicle is ≤10 mm in diameter:
	i. Start to count from outer ovarian margin of the sweep to the opposite margin ii. Consider every round or oval transonic structure within the ovarian margins to be a follicle iii. Repeat the procedure with the contralateral ovary iv. Combine the number of follicles in each ovary to obtain the AFC
	B. If the largest follicle is > 10 mm in diameter:
	i. Further ascertain the size range of the follicles by measuring each sequentially smaller follicle, in turn, until a follicle with a diameter of ≤10 mm is found ii. Perform a total count (as described) regardless of follicle diameter iii. Subtract the number of follicles of > 10 mm from the total follicle count

2010 [13])

AFC = antral follicle count.

**Figure 2:**
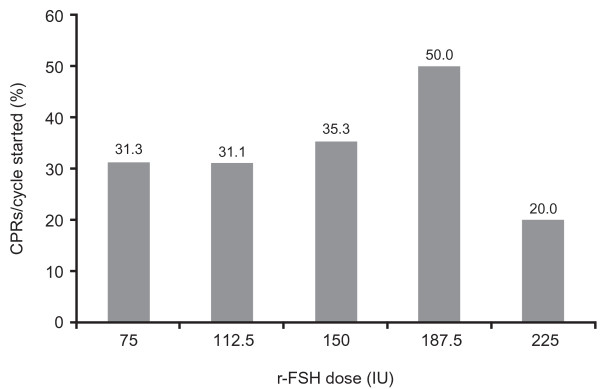
**Treatment strategies and predicted response to COS based on patient circulating AMH levels**. Pregnancy outcomes after COS with r-FSH doses determined by basal FSH, BMI, age and AFC using the CONSORT dose calculator algorithm. AFC = antral follicle count; FSH = follicle-stimulating hormone; ASN = asparagine; Ser^680 ^= serine^680^; BMI = body mass index; CONSORT = Consolidated Standards of Reporting Trials; COS = controlled ovarian stimulation; r-FSH = recombinant follicle-stimulating hormone. Adapted from Olivennes et al. 2009 [14].

Similarly to AMH, AFC was initially used as a simple prognostic factor and subsequently become a decision-making variable. Following the initial establishment of AFC as a reliable prognostic factor, its use in matching patients with COS protocols was hypothesised. A first attempt to address this was the Consolidated Standards of Reporting Trials (CONSORT) analysis, which aimed to determine whether specific factors could optimally predict a response to stimulation in ART and then develop a treatment algorithm to determine the optimal starting dose of r-FSH [14]. Results from a subsequent CONSORT study demonstrate that AFC, combined with other patient characteristics, can be used to personalise treatment and maximise COS response and, at the same time, minimise patient risk and treatment burden [15]. The CONSORT dosing algorithm was used to calculate the dose of r-FSH with 37.5 IU incremental increases based on basal FSH, body mass index (BMI), age and AFC. Patients were assigned to r-FSH doses of 75 IU (n = 48), 112.5 IU (n = 45), 150 IU (n = 34), 187.5 IU (n = 24) and 225 IU (n = 10). Oocytes retrieved per cycle were 8.5, 8.0, 10.0, 12.0 and 8.0, and CPRs per cycle of 31.3%, 31.1%, 35.3%, 50.0% and 20.0%, respectively, were observed [15]. The favourable retrieval and pregnancy rates seen in this preliminary study demonstrate the successful use of multiple biomarkers, including AMH, to achieve effective individualised COS (Figure 2).

### Genetic biomarkers

The influence of genetic research continues to shape the scientific and medical landscape. Pharmacogenetics is the science of predicting drug effects based on the genotype of an individual. In the future, the treatment of a patient could be based on her individual DNA. Presently, even though the biological response to any given drug may be influenced by hundreds of genes, progress is being made in the identification of specific genetic variances, called single nucleotide polymorphisms (SNPs) that can predict the safety and effectiveness of certain drugs in individual patients. In the field of reproductive health, hormonal and functional biomarkers are more established as tools to predict ovarian response, but in the future, genetic biomarkers may well be the best predictive tool to guide individualised treatment.

Genetic traits that influence fertility may not have visible clinical signs or abnormalities. If a patient's genetic profile also diminishes her response to fertility treatment, the failure to consider the genotype when designing the treatment consequently leads to a suboptimal treatment strategy. For example, a subset of young, normogonadotrophic IVF patients may produce an adequate number of retrieved oocytes and normal oestrogen levels, but not respond to COS as anticipated. These women require high doses of FSH (> 2,500 IU) over long treatment periods and, despite good prognostic indicators, have low implantation and pregnancy rates [16-19]. One possible reason for this hyporesponder 'normal' population is that they may have a genetic predisposition to a reduced sensitivity to FSH.

FSH and LH synergistically regulate normal ovarian function and oocyte development, and the disruption of either hormonal signal could have an impact on normal ovarian function and stimulation response. Thus, the genetic variability that affects the innate activity of these hormones could provide valuable predictive information and help guide COS treatment choices. Mutations in the genes coding for LH [20-24], the LH receptor [25-27] and the FSH receptor [28-32] have been identified as possible causes of subfertility, as well as factors that may influence fertility treatment.

**Figure 3:**
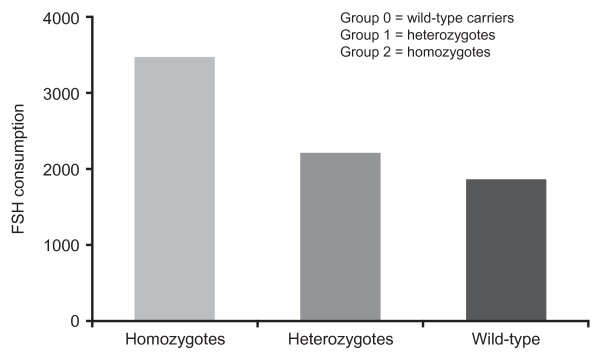
**Association between v-LH and FSH consumption**. FSH = follicle-stimulating hormone; v-LH = variant luteinising hormone. Adapted from Alviggi et al. 2009 [34].

A common variant of the β subunit of the LH molecule (v-LH) is identified by an additional sulphonated sugar at asparagine (Asn)-13 [33]. The v-LH polymorphism is found in populations worldwide, but has so far been most commonly identified in northern European countries [34]. There is clear clinical evidence that the v-LH polymorphism affects FSH sensitivity and the ovarian response to COS. For example, in a group of 60 normogonadotrophic women aged 18-37 years with normal menstruation, basal FSH ≤10 IU/l and at least five oocytes retrieved, one homozygote and seven v-LH heterozygotes were identified. When these women were stratified by the cumulative dose of FSH used (> 3,500 IU, 2,000-3,500 IU or < 2,000 IU), one heterozygote fell in the middle range with the rest having had doses greater than 3,500 IU [24]. In another study of 204 normogonadotrophic women, 21 v-LH heterozygotes and three v-LH homozygotes were identified. The FSH dose needed was positively correlated with the presence of the mutation, with a gradient in which the v-LH homozygotes and heterozygotes received a substantially higher cumulative dose when compared with the wild-type carriers (Figure 3) [35]. These preliminary analyses suggest that the v-LH genotype may help identify a subgroup of potential COS hyporesponders who are less sensitive to FSH, and who would benefit from LH supplementation rather than stimulation with higher doses of FSH.

**Figure 4:**
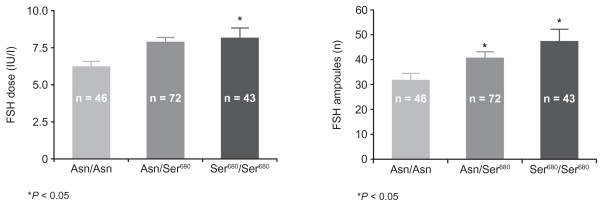
**Basal FSH levels and ampoules of FSH used in COS for patients with variants of the FSH receptor**. Basal FSH levels (left panel) and ampoules of FSH used in COS (right panel) for homozygote wild-type (Asn/Asn), heterozygote (Asn/Ser^680^) and homozygote (Ser^680^/Ser^680^) carriers of the Ser^680 ^variant of the FSH receptor. ASN = asparagine; CPRs = clinical pregnancy rates; COS = controlled ovarian stimulation; FSH = follicle-stimulating hormone; r-FSH = recombinant follicle-stimulating hormone; Ser^680 ^= Serine^680^. Adapted from Perez Mayorga et al. 2000 [37].

Hyposensitivity to FSH may also be caused by a genetic variant of the FSH receptor. Two FSH receptor variants that have SNPs in the coding region have been identified and well characterised [36]. The SNP known as the Serine^680 ^(Ser^680^) variant causes the replacement of Asn with Ser at the 680 position, which is located in the intracellular domain of the FSH receptor protein [37]. Consistent with reduced sensitivity to endogenous FSH, carriers of this trait have higher FSH levels throughout most of the menstrual cycle, and a significant increase in both total menstrual cycle length and number of antral follicles [29]. When this SNP was studied in a group of 161 women below the age of 40 years undergoing ART, the distribution was 45% for the wild-type (Asn/Asn), 29% for the heterozygote (Asn/Ser^680^) and 26% for the homozygote (Ser^680^/Ser^680^). Although peak oestradiol levels, numbers of pre-ovulatory follicles and numbers of retrieved oocytes were similar in the three groups, basal FSH levels were significantly higher for carriers of the Ser^680 ^variant (6.4 IU/l, 7.9 IU/I and 8.3 IU/l for the Asn/Asn, Asn/Ser^680 ^and Ser^680^/Ser^680 ^groups, respectively). Furthermore, both the heterozygotes and homozygotes required significantly more FSH during COS compared with the wild-type group (Figure 4) [38]. The second receptor variant, known as the Alanine^307 ^(Ala^307^) variant, is generated through substitution of threonine (Thr) with Ala at the 307 position, located in the extracellular domain of the FSH receptor [37]. There is a very strong linkage disequilibrium between the two SNPs. This means that women who possess Thr^307 ^nearly always have Asn^680 ^present on the same allele, and women who have Ala^307 ^have Ser^680 ^on the same allele [36]. The link between FSH receptor SNPs and polycystic ovarian stimulation (PCOS) has been studied extensively; however, there is some variation in the results seen. Some studies have demonstrated higher levels of patients with Ser^680 ^in the PCOS population [39], whereas other investigators have shown differences in basal levels of FSH depending on the presence of the SNP [40]. Interestingly, it has recently been demonstrated that PCOS patients with the Ser^680 ^mutation have a natural resistance to clomiphene citrate [41]. This may prove to be very important in the field of ovarian stimulation in the future with clinicians devising their COS protocols according to factors including FSH receptor genotype. Additionally, the effect of the Ser^680 ^SNP has been studied in the general population receiving subfertility treatment and results have been reviewed previously [42]. Studies have reported conflicting results with some demonstrating worse outcomes with the mutation and others demonstrating positive outcomes. For example one study demonstrated higher pregnancy rates in the patients with Asn680 [43] and another showed higher pregnancy rates in patients with Ser680 [44].

An additional study looked at FSH-induced oestradiol levels in women who were homozygous for the Ser^680 ^variant compared with women with the wild-type (Asn/Asn). This represented the first attempt to develop a pharmacogenomical approach to COS. Ser^680^/Ser^680 ^carriers were randomly allocated to two subgroups to receive a daily FSH dose of 150 IU or 225 IU, respectively. Age and BMI-matched Asn/Asn carriers, receiving a daily dose of 150 IU, constituted a third (control) group. Even though the treatment details, number of oocytes retrieved and fertilisation rates were similar, the wild-type group had higher oestradiol production after treatment with 150 IU/day of FSH compared with the Ser^680 ^group given the same dose; conversely, when Ser^680^/Ser^680 ^carriers were treated with 225 IU/day, this difference disappeared [31]. These findings are consistent with carriers of the Ser^680^/Ser^680 ^variant experiencing lower biological activity of both endogenous and exogenous FSH due to lower sensitivity of the FSH receptor to FSH. However, it should be noted that two further studies did not confirm that adjustment of the starting dose of FSH improved the hormonal response [43,44]. Despite this, the findings of FSH starting dose modification studies raise an intriguing question about the interpretation of isolated markers, including AFC. In fact, the current literature suggests that AFC is effective in predicting ovarian response and choosing the right FSH dose; nevertheless, a pharmacogenomical approach to COS strongly indicates that, in at least one out of four patients with normal AFC (which reflects the frequency of Ser^680^/Ser^680^), the optimal FSH dose should be higher. This provides further support that AFC and genetic approaches should be integrated into determination of COS treatment protocols.

In addition to the two SNPs described above, there are also many genotypic SNPs found in the introns and untranslated regions of the FSH receptor gene; however, it is not currently known what physiological effects these mutations confer [36]. Further mutations have been identified in young women (< 35 years) undergoing COS in the form of receptor splice variants [32]. FSH receptor messenger RNA was isolated from the cumulus cells of women who demonstrated high or low response to FSH when treated with a GnRH agonist long protocol. Abnormal splicing products were identified that affected the ligand-binding extracellular domain. One variant was associated with a low response to FSH, and another variant was associated with a high FSH response [32].

### Patient profiling

Each patient's individual characteristics must be considered in order to determine treatment that will provide her with an optimal response to COS, while keeping the adverse events and treatment burden as low as possible. Specifically, the hormonal, functional and genetic biomarkers described here can provide the critical information needed to personalise treatment to best serve the individual patient. AMH represents ovarian reserve and can help determine how much longer a woman will remain fertile and if ART is a viable option. AFC represents the number of follicles that are maturing in each cycle and provides a prediction of the response to COS. Finally, the individual patient's genetic profile defines the underlying physiology that determines the meaning and interpretation of hormonal and functional biomarkers like AMH and AFC, predicting the effectiveness of FSH when stimulating the ovaries (Table 3).

**Table 3:** Patient genetic profiles: interpretation of physiology and biomarker levels

Genetic profile	Interpretation of genetic profile
Low AMH levels; low AFC	Suggests that even high doses of FSH would be ineffective and that LH would not improve results Patient would benefit from counselling to better understand her limited chances of success
FSH receptor variant (eg. Ser^680^); good AMH levels; good AFC	Suggests a good prognosis, but it also predicts a genetic hyposensitivity to FSH that should be considered when formulating COS treatment
v-LH (variant in β subunit of LH receptor)	Suggest that a patient might benefit from LH supplementation during COS

AMH = anti-Müllerian hormone; AFC = antral follicle count.

In addition to using the patient's individual characteristics to determine which treatment protocols should be selected, this patient-specific approach could potentially be used to determine how treatment protocols are administered. The short, ultrashort, micro-flare and stop GnRH agonist protocols are all adaptations of the standard protocol for COS, generated by adjusting the timing and dose of GnRH agonist administration [45,46]. Preparation of the endometrium is also a crucial step in the success of IVF. Further analysis of hormonal, functional and genetic biomarkers and their relationships with patient response to variations in treatment protocols could be used in the future, and additional clinical data are required to support this hypothesis.

## Conclusions

Individual patient characteristics can be predictive of ovarian response, and these factors must be used to optimise the individual treatment regimen. AMH and AFC are good predictors of ovarian response to COS, and may be valuable in deciding which protocol and dose to use for COS. Additionally, initial data indicate that the use of AMH to determine protocol selection may reduce the incidence of OHSS, although further studies are required to confirm this. The hyposensitivity to FSH in a subset of women undergoing the GnRH agonist protocol despite other normal indicators was described by De Placido and colleagues [17]. It is possible that this condition may be caused by genetic polymorphisms of LH, the FSH receptor or the LH receptor. Thus, genetic testing of normogonadotrophic women showing reduced sensitivity to FSH (e.g., a high FSH dose in previous cycles) may assist in tailoring subsequent treatment. Patients could be given higher doses of FSH if they are found to carry the Ser^680 ^variant, or have LH added to their treatment if they are found to carry the v-LH polymorphism. As technology progresses and more powerful analytical tools are developed, predicting ovarian response using a single biomarker will not be sufficient to make the most accurate prognosis or to formulate a personalised treatment plan. In the future, the combination of hormonal, functional and genetic testing will be needed to ensure that the right treatment protocol is provided to the right patient. Finally, the patient's response to treatment should be monitored closely to determine if further tailoring of the personalised treatment plan is required.

## List of abbreviations

AFC: antral follicle count; Ala: alanine; AMH: anti-Müllerian hormone; ART: assisted reproductive technology; Asn: asparagine; BMI: body mass index; CONSORT: Consolidated Standards of Reporting Trials; COS: controlled ovarian stimulation; CPR: clinical pregnancy rate; DHEA: dehydroepiandrosterone; DNA: deoxyribonucleic acid; FSH: follicle-stimulating hormone; GnRH: gonadotrophin-releasing hormone; hCG: human chorionic gonadotrophin; hMG: human menopausal gonadotrophin; IVF: in-vitro fertilisation; LH: luteinising hormone; OHSS: ovarian hyperstimulation syndrome; PCOS: polycystic ovary syndrome; r-FSH: recombinant follicle-stimulating hormone; r-hCG: recombinant human chorionic gonadotrophin; r-hLH: recombinant human luteinising hormone; RNA: ribonucleic acid; Ser: serine; SNP: single nucleotide polymorphism; Thr: threonine; u-FSH: urinary follicle-stimulating hormone; u-hCG: urinary human chorionic gonadotrophin; v-LH: variant luteinising hormone

## Competing interests

CA and PH have no competing interests to declare. DE is an employee of Merck Serono S.A., Geneva.

## Authors' contributions

CA, PH and DE all contributed their opinions and were involved in the reviewing of the literature and writing of the manuscript. All authors read and approved the final manuscript.
